# Effect of fundamental frequency removal on mistuned harmonic separation thresholds

**DOI:** 10.3389/fpsyg.2026.1814083

**Published:** 2026-06-10

**Authors:** Christel Azar, Alexis Whittom, Loonan Chauvette, Florence Couture, Claudelle Nadeau, Benoit-Antoine Bacon, François Champoux, Andréanne Sharp

**Affiliations:** 1 École d’orthophonie et d’audiologie, Faculté de médecine, Université de Montréal, Montréal, QC, Canada; 2 Centre de recherche de l’Institut Universitaire de Gériatrie de Montréal, Montréal, QC, Canada; 3 École des sciences de la réadaptation, Faculté de médecine, Université Laval, Québec City, QC, Canada; 4 CERVO Brain Research Center, Université Laval, Québec City, QC, Canada; 5 Department of Psychology, The University of British Columbia, Vancouver, BC, Canada

**Keywords:** harmonic separation, missing fundamental, mistuned harmonic, pitch perception, sound segregation

## Abstract

**Introduction:**

Investigating how the auditory system processes harmonics is essential for advancing our understanding of sound segregation mechanisms and for guiding the development of technologies that support speech and music perception in complex listening environments, particularly for aging populations and individuals with hearing loss. Previous studies have investigated the extent of mistuning required for a harmonic to be perceived as distinct from a complex tone and how factors like modulation patterns, onset timing and ear of presentation can influence this threshold. However, the effect of a missing fundamental frequency on mistuned harmonic separation thresholds remains unexplored.

**Method:**

This pilot study, conducted using an adjustment method developed in our lab for this specific application, investigated mistuning thresholds for the first harmonic in complex tones with and without F_0_ at 125, 200 and 440 Hz.

**Results:**

Because the F_0_ helps define the harmonic structure of a sound, its removal was expected to impair the perceptual framework used to detect pitch deviations, thereby increasing mistuning thresholds, as predicted by current models of pitch perception. Contrary to expectations, the results revealed a significant overall improvement in performance when the F_0_ was missing.

**Discussion:**

These results have important implications for understanding pitch processing as it suggests that the auditory system may more effectively detect pitch irregularities by relying on the harmonic structure of a sound rather than on the presence, or relationship with, its fundamental frequency.

## Introduction

In most situations, it is rare to be exposed to isolated pure sounds. Humans are much more likely to be hearing complex sounds, often from multiple sound sources, with various temporal and spectral characteristics. The auditory system then needs to decompose, analyze, and assemble auditory information into perceptually meaningful mental representations, a process known as “auditory scene analysis” (Bregman, 1994). Pitch discrepancies play a crucial role in assisting listeners to effectively separate and comprehend competing sound sources within complex acoustic environments. Indeed, most sounds encountered in human environment are complex periodic tones with multiple harmonics that tend to be perceived as a unified entity with a pitch closely resembling the fundamental frequency (F_0_) of the tone (Darwin, 2005). These stimuli have been the primary focus of most studies on pitch perception due to their significance in speech and music perception (Plack et al., 2005).

The amount of mistuning needed for a harmonic to be perceived as a distinct entity from a complex tone have already been revealed (Moore et al., 1986). Experiments surrounding mistuned harmonics have also examined how factors such as frequency or amplitude modulation patterns, onset asynchrony, and ear of presentation influence the perceptual segregation of the mistuned component from the harmonic complex (Darwin and Ciocca, 1992; Darwin et al., 1994). Harmonic number plays a crucial role in how the auditory system segregates concurrent sounds. Lower-numbered harmonics are particularly important for sound segregation, as they are more perceptually salient and lead to better performance in mistuning and stream segregation tasks, reflected in lower detection thresholds (Alain and McDonald, 2007; Zendel and Alain, 2013). In contrast, higher-numbered harmonics are more difficult to process, especially for listeners with hearing impairment (Bianchi et al., 2019). In addition to acoustic factors, recent findings suggest that mistuning can enhance the perceptual salience of a harmonic, engaging auditory attention and facilitating the detection of concurrent sound objects (Koulaguina et al., 2015). However, none of these experiments have investigated the impact of a missing F_0_ on mistuned harmonic separation thresholds.

Pitch plays an important role in speech in noise recognition, especially in conditions involving competing speech (Vestergaard and Patterson, 2009), with increasing differences in F_0_ leading to better discrimination of concurrent vowels (Culling and Darwin, 1993) and sentences (Bird et al., 1998). It has been shown repeatedly that it is possible to completely remove the F_0_ of a stimulus without affecting pitch perception, a phenomenon called the “missing F_0_” (Seebeck, 1841; Schouten, 1938). This phenomenon helps to ensure that the identity of a sound is preserved even when it is partially masked (e.g., Licklider, 1956; McDermott and Oxenham, 2008). However, the extent to which the F_0_ is important in the integration of the other harmonic frequencies toward forming a unique complex tone remains undetermined. Given its central role in pitch perception, one would expect that the mistuned separation threshold would increase with the degradation of a complex harmonic signal caused by the removal of its primary component. The objective of this pilot study, conducted using an adjustment method designed for this specific experimental context, was to examine the impact of a missing F_0_ on mistuned harmonic separation thresholds.

## Materials and methods

### Participants

Twenty-four adults (8 males) aged 26 ± 5 years (range: 21–38), with 6 years or less of musical or combined dance and music experience (Zhang et al., 2020) were recruited. All participants had normal hearing confirmed by audiometric screening. Thresholds from 0.25 to 8 kHz were measured in both ears in a quiet room and were considered normal if below 25 dB HL (Kamerer et al., 2022). The Research Committee for sectorial research in neurosciences and mental health of the CIUSSS - Capitale Nationale approved all procedures, and each participant provided written informed consent. All experiments were performed in accordance with relevant guidelines and regulations.

### Rationale for sample size

Previous studies in the field have used small samples (e.g., Hartmann et al., 1990: *n* = 6; Alain et al., 2001: *n* = 12; Roberts and Holmes, 2006: *n* = 10) and reported statistically significant effects, suggesting large effect sizes. Based on this, we selected a moderate effect size (*f* = 0.25) for an *a priori* power analysis using G*Power v.3.1.9.7 (Faul et al., 2007). For a 2 × 3 repeated-measures ANOVA, the analysis indicated that a sample size of 19 participants would be sufficient to detect medium effects (*f* = 0.25) with a power of 1 − *β* = 0.80 and a significance threshold of *α* = 0.05, assuming sphericity and a 0.5 correlation between repeated measures. Our final sample of 24 exceeds that estimation and the sample sizes typically used in prior studies.

### Stimuli and procedure

**Figure 1:**
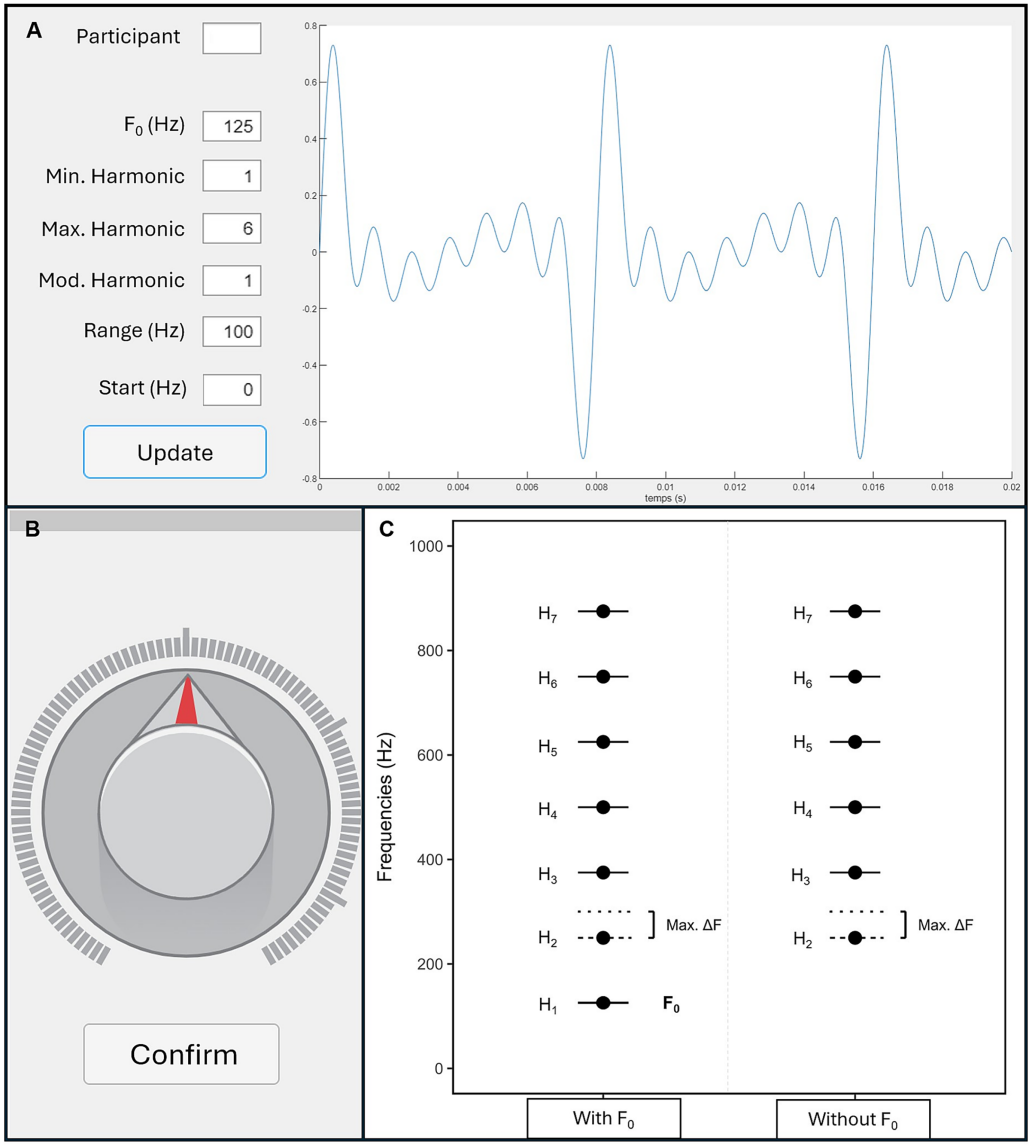
Harmonicity MATLAB-based app interfaces. **(A)** Experimenter interface, **(B)** participant interface, and **(C)** graphical representation of the generated sound and task with and without F₀.

Using a MATLAB-based application developed in our laboratory (see Figure 1A), all participants had to progressively mistune the frequency of the first (lowest) harmonic (H_2_) of a complex sound comprising 6 harmonics (H_2_-H_7_) of equal amplitude until they reached separation threshold. Trials were fully randomized across F_0_ frequencies and F₀ conditions (with and without F_0_), with five repetitions per frequency–condition combination, for a total of 30 trials. The harmonic complex tones were presented to the participants through headphones (Insert ER3C, Etymotic) at a comfortable sound level (65-70 dB SPL) in a quiet room. The sound level was calibrated at the beginning of the experiment and remained fixed throughout all trials. No adjustments were made between conditions with or without the F_0_, as output measurements confirmed that the overall levels were comparable across stimuli. The output levels were measured using a Brüel & Kjær Type 2,250 sound level meter equipped with a pressure-field microphone and a click-on coupler adapted for insert earphones. Participants were instructed to use the computer mouse to move the arrow of a digital dial (see Figure 1B) exclusively upward to mistune H_2_ until they heard two distinct sounds (downward answers were discarded and trial was resumed). The maximum mistuning allowed by the dial was 50 Hz (see Figure 1C). Furthermore, they were told that a beating pattern (Plack, 2010) did not correspond to the desired perception. Here, a beating pattern refers to the perceptual experience resulting from slow, periodic fluctuations in sound amplitude resulting from interference between closely spaced frequency components, producing a percept of roughness rather than a stable, fused sound. Participants were instructed to disregard such beating patterns and instead to attend to situations in which a sound component perceptually “popped out,” that is, became clearly segregated from the complex due to mistuning. A practice test with instructions and examples was performed using speakers. After becoming acquainted with the task, they had to complete the rest of the experiment with closed eyes to avoid any reliance on visual cues in adjusting the dial to the same spot on each trial. The starting point on the dial was randomized for each trial (middle left, center, and middle right) to prevent the participant from repeating the same movement with the mouse. The task was repeated using three different complex sounds, each with a distinct fundamental component (F₀: 125 Hz, 200 Hz, and 440 Hz) and their related harmonics, presented in random order under two conditions: with and without F₀. The selected fundamental frequencies were chosen to sample a pitch range commonly used in psychoacoustic studies of harmonic mistuning (e.g., Hartmann et al., 1990; Moore et al., 1986), where harmonics are well resolved and pitch perception is reliable (approximately 100–500 Hz). Within this range, 440 Hz corresponds to the standard musical reference pitch (A4), providing a familiar sound for many participants, while the lower values (125 Hz and 200 Hz) extend the stimuli to pitch regions more typical of speech and low-frequency musical sounds. Although some of these frequencies lie closer to conventional musical note values than others, the intent was not to contrast musical versus non-musical tones, but rather to demonstrate that the observed effects were not restricted to a single pitch region.

### Analysis

For all conditions of frequencies and present/missing F_0_, intra-class correlation coefficients (ICC) were computed to estimate test–retest reliability across trials (Koo and Li, 2016). For each participant and condition, the average of the 5 trials was computed to determine the mistuned harmonic separation thresholds (Δf) when the F_0_ was present and missing. The Δf was expressed as a percentage deviation from harmonicity relative to the frequency of the harmonic:


$$(\frac{\text{Amount of mistuning}}{F_{0}\times \text{Harmonic number}})\times 100$$

Repeated measures ANOVA were carried out to determine whether there was a significant difference between Δf for each frequency, with and without F_0_.

## Results

ICC estimates and their 95% confident intervals were calculated using R (v4.4.0) and the *irr* package (v0.84.1) based on a mean-rating (k = 5), absolute-agreement, 2-way mixed-effects model and interpreted according to the guidelines from Koo and Li (2016). The test–retest reliability of the repeated thresholds task was found to range from moderate to excellent across all conditions (Table 1).

**Table 1:** ICC results for all conditions.

Conditions	ICC value	95% Confidence interval	Interpretation
125 Hz without F_0_	0.79	[0.61–0.90]	moderate to good
200 Hz without F_0_	0.86	[0.75–0.93]	moderate to excellent
440 Hz without F_0_	0.95	[0.90–0.97]	excellent
125 Hz with F_0_	0.84	[0.71–0.92]	moderate to excellent
200 Hz with F_0_	0.72	[0.49–0.86]	moderate to good
440 Hz with F_0_	0.90	[0.82–0.95]	good to excellent

A two-way repeated-measures ANOVA was conducted using R (v4.4.0) and the *rstatix* package (v0.7.2) to examine the effects of F_0_ condition (present, absent) and frequency condition (125, 200, 440 Hz) on Δf thresholds. Univariate outliers and extreme points were identified for each condition using Tukey’s fence method (Tukey, 1977) with factors of 1.5 IQR and 3 IQR, respectively. Six outlier values and one extreme point were identified across five participants and were removed for the main analysis but were included in sensitivity analysis. Univariate normality was assessed using Shapiro–Wilk tests and Q–Q plots, with results indicating a normal distribution in all but one condition (*p* > 0.05) and visual inspection supporting normality.

**Figure 2:**
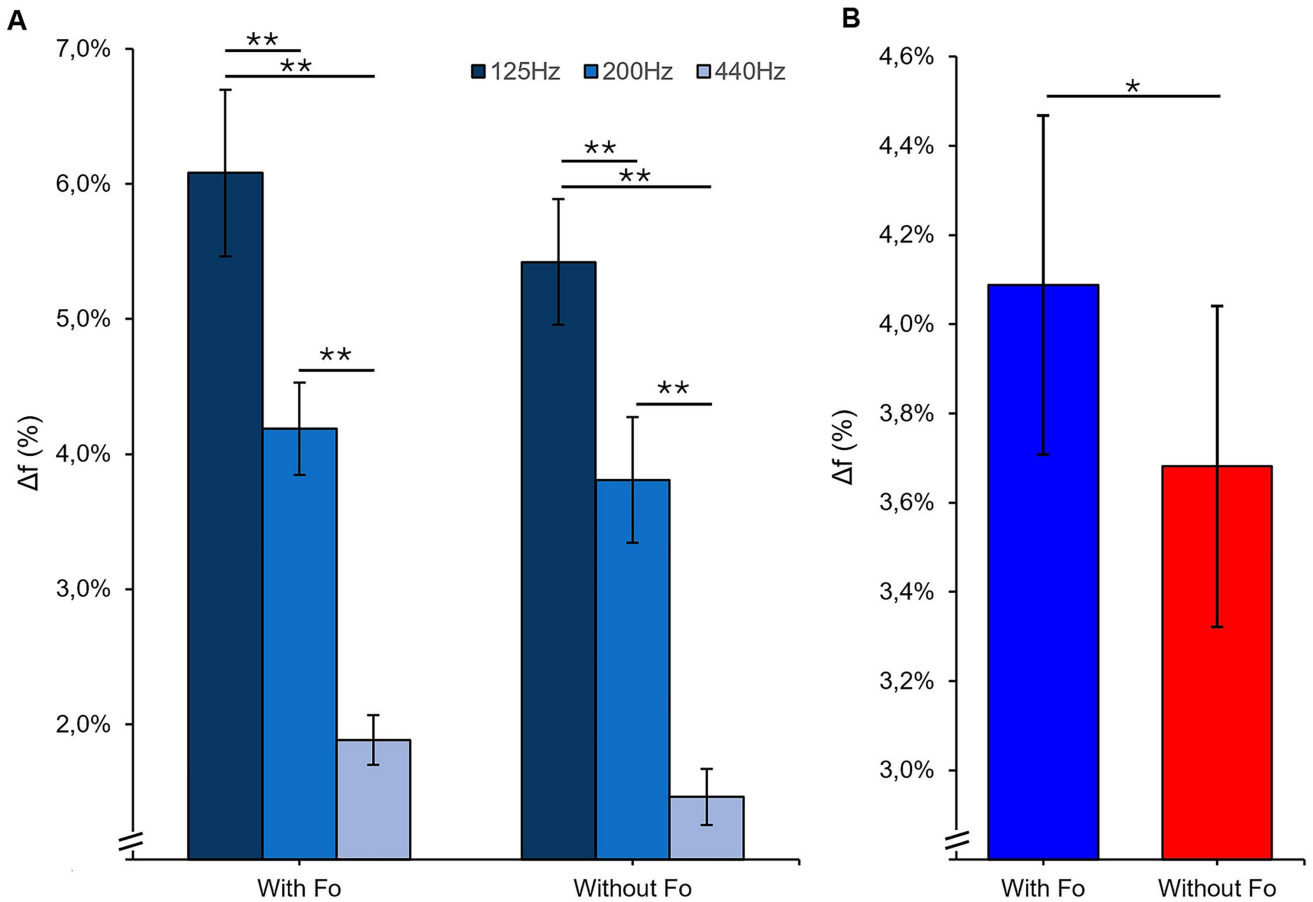
Thresholds of mistuning required to perceive the lowest harmonic (H_2_) as out of tune. **(A)** Comparison across frequencies with and without F_0_. **(B)** Average thresholds across all frequencies, comparing conditions with and without F_0_. Error bars represent SEM. *p < 0.05, **p < 0.001.

The ANOVA revealed a significant main effect of frequency condition, *F*(2, 38) = 104.27, *p* < 0.001, η^2^_G_ = 0.566, indicating differing Δf thresholds across frequencies (Figure 2A). A significant main effect of F_0_ condition was also observed, *F*(1, 19) = 10.75, *p* = 0.004, η^2^_G_ = 0.032, indicating higher Δf thresholds when F_0_ was present (Figure 2B). The interaction between F_0_ condition and frequency was not significant, *F*(2, 38) = 0.22, *p* = 0.806, η^2^_G_ = 0.001. A sensitivity analysis including outliers yielded consistent results, with both main effects remaining significant while the interaction effect remained nonsignificant. Post-hoc paired-samples *t* tests with Holm correction were conducted on the entire sample, revealing significantly higher Δf thresholds for 125 Hz (M = 5.87, SD = 2.67) compared to 200 Hz (M = 4.06, SD = 1.82), *t*(47) = 4.16, *p* < 0.001 and 400 Hz (M = 1.73, SD = 0.92), *t*(47) = 11.61, *p* < 0.001, and for 200 Hz compared to 400 Hz, *t*(47) = 7.47, *p* < 0.001.

## Discussion

The primary aim of this study was to investigate the impact of a missing F_0_ on mistuned harmonic separation thresholds. Although several studies have examined mistuning detection in complex tones with a present fundamental frequency (F_0_; e.g., Hartmann et al., 1990; Alain et al., 2001; Roberts and Holmes, 2006; Faul et al., 2007; Plack, 2010; Koo and Li, 2016; Tukey, 1977), direct comparisons with our thresholds are difficult due to methodological differences. Hartmann et al. (1990) used a pitch-matching task with musically trained co-authors, focusing on pitch shift estimation rather than perceptual segregation, and allowing reliance on cues like beating and roughness. Alain et al. (2001) employed a two-alternative forced-choice procedure that likely captured general inharmonicity detection using similar cues. Roberts and Holmes (2006) inferred grouping strength from pitch shifts rather than measuring mistuning thresholds directly. These differences in task design and participant expertise likely account for the lower thresholds reported in those studies. In contrast, our adjustment method was designed to measure the mistuning required for perceptual segregation of a component tone, minimizing reliance on beating cues. This approach aligns with Moore et al. (1986), whose similar perceptual instructions that required listeners to adjust harmonic mistuning until a component was perceived as a separate sound yielded comparable results. Indeed, mistuning thresholds for H_2_ with F_0_ at 125, 200, and 440 Hz in our study fell within the range reported by Moore et al. (1986) for 100, 200, and 400 Hz, supporting the validity of our paradigm. Results also revealed a significant frequency effect in mistuning thresholds, with higher thresholds observed at 125 Hz compared to 200 Hz and 440 Hz, regardless of F_0_ presence. This trend is consistent with the findings of Moore et al. (1986), who observed similar patterns in experiments using complex tones at comparable frequencies. A possible explanation is that, at lower fundamental frequencies, the spacing between adjacent harmonics is reduced, leading to greater overlap within the auditory filters (Smith and Abel, 1999). This overlap diminishes the distinct representation of harmonics, making mistuning harder to detect. In such cases, harmonics are often unresolved, and masking effects become more pronounced due to the proximity of harmonics within the auditory filters, as described by the concept of harmonic resolvability (Moore et al., 1985; Moore and Gockel, 2011; Bianchi et al., 2016; Whittom et al., 2023). Finally, and most importantly, this study shows that, counterintuitively, mistuned harmonic separation thresholds were significantly lower in all conditions when F_0_ was absent.

The overall improved thresholds observed when F_0_ is absent may be explained by several factors. One potential explanation is that removing the F_0_ simplifies the harmonic interactions within the complex tone, making the separation of individual harmonics more perceptible. This could enhance the listener’s ability to rely on alternative auditory cues, such as harmonic spacing, namely the regular interval between harmonics, a cue that has been shown to facilitate pitch discrimination in the absence of the F_0_ (Cariani and Delgutte, 1996; de Cheveigné and Pressnitzer, 2006; Ladd et al., 2013).

Most models that attempt to explain the perception of the missing F_0_ or mistuned harmonic perception are mainly based on 3 theories who share a common concept of central formation of pitch (Goldstein, 1973; Terhardt, 1974; Wightman, 1973). Behavioral and electrophysiological experiments support that the missing F_0_ phenomenon originates from central processing (Licklider, 1956; Coffey et al., 2016; Houtsma and Goldstein, 1972; Zatorre, 1988). Experiments from Lin and Hartmann (1998) also suggest that mistuned harmonic perception rely on a central template. In the present study, the improved separation thresholds observed when F_0_ is absent may reflect reduced perceptual fusion, allowing the mistuned partial to stand out more clearly within the harmonic complex, allowing the brain to adapt the central template to more efficiently process harmonic relationships. This aligns with the idea that the brain’s internal model could be sensitive to the spectral configuration of the stimulus and may adaptively adjust grouping strategies depending on the presence or absence of F_0_. These findings point to the importance of central processing in harmonic segregation and warrant further investigation into how internal models interact with stimulus structure in both missing fundamental and mistuned harmonic perception. Alternatively, changes in the spectral energy distribution introduced by the presence of the F_0_ may alter the spectral energy distribution by activating low-frequency auditory filters, potentially reducing the relative salience of the second harmonic (H_2_). It may also eliminate spectral edge effects, thereby making it less perceptually distinct within the complex tone. There is evidence suggesting that spectral-edge components are more perceptually salient and resolvable than internal components due to the lack of masking on one side (e.g., Dai, 2010; Kohlrausch and Houtsma, 1992). In line with these findings, one hypothesis that could help explain the observed pattern is that the target’s shift from a masked, internal position to a highly salient spectral-edge position increased its perceptual salience, although the underlying mechanism is not yet clearly established. These interpretations are consistent with known auditory processing mechanisms and offer plausible explanations for the observed pattern. Although our study was not designed to isolate these mechanisms, they provide valuable directions for future investigation.

In designing the experiment, the decision to mistune the second harmonic (H_2_), rather than higher harmonics such as H_3_ or H_4_, was guided by its perceptual proximity to the fundamental frequency (F_0_), making it the most suitable choice for isolating the perceptual contribution of F_0_ presence or absence. Mistuning thresholds vary nonlinearly with harmonic rank (Hartmann et al., 1990), and higher-order harmonics tend to engage distinct auditory grouping mechanisms. Moreover, mistuning H_2_ is associated with relatively minor timbre changes compared to higher harmonics (such as H_3_), which more substantially affect the spectral envelope and brightness (Siedenburg et al., 2021; McAdams and Siedenburg, 2019). While any mistuning may introduce some degree of timbre variation, the choice of H_2_ allowed us to probe F_0_-related effects with minimal confounding from timbre-related cues, therefore aligning with the core objective of the study.

Still, it should be acknowledged that the present design does not allow us to disentangle the effects of F_0_ presence/absence from those of harmonic adjacency, since the presence of F_0_ also introduces an additional adjacent harmonic (H_1_) near the mistuned H_2_. This study was not intended to isolate the effects of harmonic rank per se, but rather to examine how the presence or absence of F_0_ modulates the perceptual impact of mistuning under conditions of minimal timbral change. As such, the present findings should be interpreted within the context of H_2_ mistuning. Future studies could explore whether similar effects are observed when mistuning other harmonics, which would help clarify whether the observed effects are driven by F_0_ presence or by the surrounding spectral context.

Finally, methods that measure the magnitude of pitch shifts induced by mistuning of harmonics (Darwin and Ciocca, 1992; Darwin et al., 1994) could also be applied to complex tones with missing fundamentals to assess if the pattern of pitch shift changes in the absence of the F_0_. However, while our methodology captures the threshold at which the mistuned partial is perceived as a separate auditory object, it does not provide insight into the perceptual experience leading up to that moment, such as whether participants perceive a gradual change in pitch, a shift in the direction of pitch, or other qualitative transformations in the sound. Investigating these perceptual dynamics could enrich our understanding of the separation process and represents a promising direction for future research.

Given the central role of pitch in speech and music perception, it is also important to consider how age-related auditory changes may affect the ability to process such complex sounds. Age-related hearing loss and changes in central auditory processing have been shown to negatively affect the ability to segregate concurrent sound sources, especially in complex auditory scenes such as speech in noise (Boncz et al., 2024; David et al., 2018). Older adults often exhibit reduced sensitivity to temporal and spectral cues that are critical for auditory scene analysis, including harmonicity and periodicity cues (Alain et al., 2014; Gallun and Best, 2020). These changes can compromise the encoding and perceptual organization of complex sounds, making it more difficult to distinguish overlapping auditory objects. Understanding how specific acoustic features facilitate or hinder sound segregation is therefore highly relevant for addressing the auditory challenges faced by aging individuals and for informing auditory rehabilitation strategies. Future studies should also explore the influence of aging and hearing loss on mistuned harmonic perception in the presence or absence of F_0_.

## Data Availability

The raw data supporting the conclusions of this article will be made available by the authors, without undue reservation.
